# Targeting glial modulation: molecular mechanisms of Polygonatum in neurological disorders

**DOI:** 10.3389/fphar.2026.1969384

**Published:** 2026-10-01

**Authors:** Zilong Liu, Yixun Luo, Chuanfeng Zheng, Xiuzhen Long, Yiqi Zhao, Zhiying Pan, Bingyang Xu, Jianfei Sun

**Affiliations:** Chemistry and Biochemistry Laboratory, Guizhou Medical University, Guiyang, Guizhou, China

**Keywords:** astrocytes, microglia, natural products, neurological diseases, oligodendrocytes, Polygonatum

## Abstract

Neurological disorders arise from interconnected pathological processes, including oxidative stress, neuroinflammation, mitochondrial dysfunction, impaired proteostasis, synaptic injury and blood brain barrier disruption. Increasing evidence indicates that glial cells are not passive bystanders in these diseases, but central regulators of neuroimmune balance, metabolic support, myelin integrity and tissue repair. *Polygonatum* spp., a traditional medicinal and edible plant, contains diverse bioactive constituents, including polysaccharides, steroidal saponins and flavonoids, which exhibit multi-target neuroprotective activities. In this Review, we summarize current evidence linking *Polygonatum* and its active compounds to the modulation of glial function and neuronal survival. *Polygonatum* polysaccharides appear to suppress microglial overactivation, attenuate TLR4/NF-κB and NLRP3 inflammatory signalling, restore redox homeostasis through NRF2-related pathways, and reduce lipid peroxidation and ferroptosis. In parallel, *Polygonatum*-derived compounds may support mitochondrial function, protein homeostasis, synaptic plasticity and neurotransmitter balance, thereby improving pathological outcomes in models of Alzheimer’s disease, Parkinson’s disease, cerebral ischaemia, depression and sleep disturbance. Evidence for direct effects on oligodendrocytes, astrocytes, oligodendrocyte precursor cells and the neurovascular unit remains limited but suggests an important area for future investigation. Standardized botanical sources, extraction procedures, pharmacokinetic analyses and high-quality clinical studies will be essential to define the therapeutic potential of Polygonatum-based interventions for neurological diseases.

## 1 Introduction

Neurological diseases comprise a heterogeneous group of disorders characterized by structural and functional abnormalities of the central nervous system (CNS) and peripheral nervous system (PNS), encompassing neurodegenerative diseases, demyelinating disorders, cerebrovascular diseases, psychiatric disorders, and neuromuscular diseases (Tegueu et al., 2013). With the acceleration of global population aging and ongoing lifestyle changes, the incidence of Alzheimer’s disease (AD), Parkinson’s disease (PD), Multiple Sclerosis (MS), stroke, depression, and other neurological disorders continues to rise, imposing an increasingly substantial burden on healthcare systems worldwide (Mahboob et al., 2024; Wei et al., 2025). According to the Global Burden of Disease study, neurological disorders have become one of the leading global health challenges, with their associated mortality, disability, and socioeconomic burden continuing to increase in parallel with population aging (Steinmetz et al., 2024).

A common hallmark of neurological diseases is the remarkable complexity of their underlying pathogenic mechanisms. Multiple pathological processes, including oxidative stress (Hassan et al., 2022; Rey et al., 2023), neuroinflammation (Giri et al., 2024; Zhang W. et al., 2023), mitochondrial dysfunction (Cheng et al., 2021; Olagunju et al., 2023), disruption of protein homeostasis (Yeming Zhang et al., 2024), impaired synaptic plasticity (Hoshina et al., 2021; Menculini et al., 2021), and blood-brain barrier dysfunction (Aryal and Patabendige, 2021; Knox et al., 2022; Patabendige and Janigro, 2023), frequently interact and collectively drive disease initiation and progression.

Glial cells have emerged as critical therapeutic targets in neurological disease research (Vishnumukkala et al., 2025). As the most abundant cell population in the CNS, glial cells not only provide structural support and metabolic regulation for neurons but also play indispensable roles in neuroimmune regulation, maintenance of synaptic plasticity, myelination, and neural repair (Greenhalgh et al., 2020). Among them, microglia, the resident immune cells of the CNS, undergo dynamic polarization toward pro-inflammatory (M1) or anti-inflammatory (M2) phenotypes in response to pathological stimuli, thereby regulating inflammatory responses, phagocytic clearance, and tissue repair (Li et al., 2021; Zong et al., 2026). Oligodendrocytes are responsible for the formation and maintenance of myelin sheaths, and their dysfunction directly contributes to demyelination and axonal injury (López-Muguruza et al., 2025). Astrocytes participate in maintaining blood-brain barrier integrity, providing metabolic and trophic support to neurons, and regulating neurotransmitter homeostasis (Pivoriūnas and Verkhratsky, 2021). In addition, recently identified glial populations, particularly oligodendrocyte precursor cells (OPCs), have been recognized as key contributors to remyelination, neural regeneration, and tissue repair (Li et al., 2025).

Currently available synthetic drugs for neurological diseases predominantly target single molecular pathways. Representative examples include donepezil, an acetylcholinesterase inhibitor (Matsunaga et al., 2015; Yaghmaei et al., 2024); memantine, an N-methyl-D-aspartate (NMDA) receptor antagonist (Matsunaga et al., 2015; Yaghmaei et al., 2024); and levodopa, a dopamine precursor used for dopamine replacement therapy (Gray et al., 2022; The Parkinson Study Group, 2004). Although these agents can alleviate clinical symptoms to varying degrees, they generally fail to halt disease progression and are frequently associated with significant adverse effects (Muleiro Alvarez et al., 2024; Qi et al., 2024). In contrast, natural products exhibit the unique advantages of multi-component, multi-target, and multi-pathway regulation, making them particularly suitable for the treatment of complex neurological disorders. Bioactive natural compounds exert broad-spectrum neuroprotective effects by modulating glial cell activation, attenuating neuroinflammation, promoting remyelination, and maintaining neuronal homeostasis (Juliet Chiamaka Muoegbunam et al., 2025). Compared with conventional single-target therapeutics, natural products are better suited to modulating the intricate pathological networks underlying neurological diseases and have therefore become an important focus of contemporary neuropharmacological research.

*Polygonatum spp*., belonging to the family Asparagaceae (formerly classified under Liliaceae), are perennial herbaceous plants widely used in traditional Chinese medicine (TCM). First documented in the Shennong Bencao Jing (Shennong’s Classic of Materia Medica), *Polygonatum* has traditionally been used to replenish qi, nourish yin, strengthen the spleen, moisten the lungs, and tonify the kidneys (Lin et al., 2024). In modern TCM practice, *Polygonatum* is commonly prescribed for the treatment or adjunctive management of qi and yin deficiency (Bai et al., 2021), spleen and stomach deficiency (Guo et al., 2025; Han et al., 2025; Zhao et al., 2024), chronic debility following prolonged illness (Qu et al., 2026; Xian et al., 2023), and age-related functional decline (Chao et al., 2025; Li et al., 2025b). It has also demonstrated considerable therapeutic potential in anti-aging interventions (Zhang et al., 2026) and the prevention and management of chronic diseases (Zhang et al., 2025; Chen et al., 2025; Si et al., 2025). Increasing evidence has highlighted the neuroprotective properties of *Polygonatum* and its bioactive constituents. *Polygonatum* polysaccharides (PSPs) alleviate oxidative stress and regulate neuroimmune homeostasis (Gao et al., 2025; Teng et al., 2023; Zhang et al., 2022a; Zhou et al., 2025); steroidal saponins suppress neuroinflammation and modulate apoptosis (Lin et al., 2024; Ye et al., 2017; Zhang et al., 2023); and flavonoids exhibit potent free radical-scavenging activity while preserving mitochondrial function (Rębas, 2025; Zhang et al., 2022a). Collectively, these multi-component, multi-target, and multi-pathway pharmacological properties confer distinct therapeutic advantages for the management of neurological diseases (Figure 1).

**FIGURE 1:**
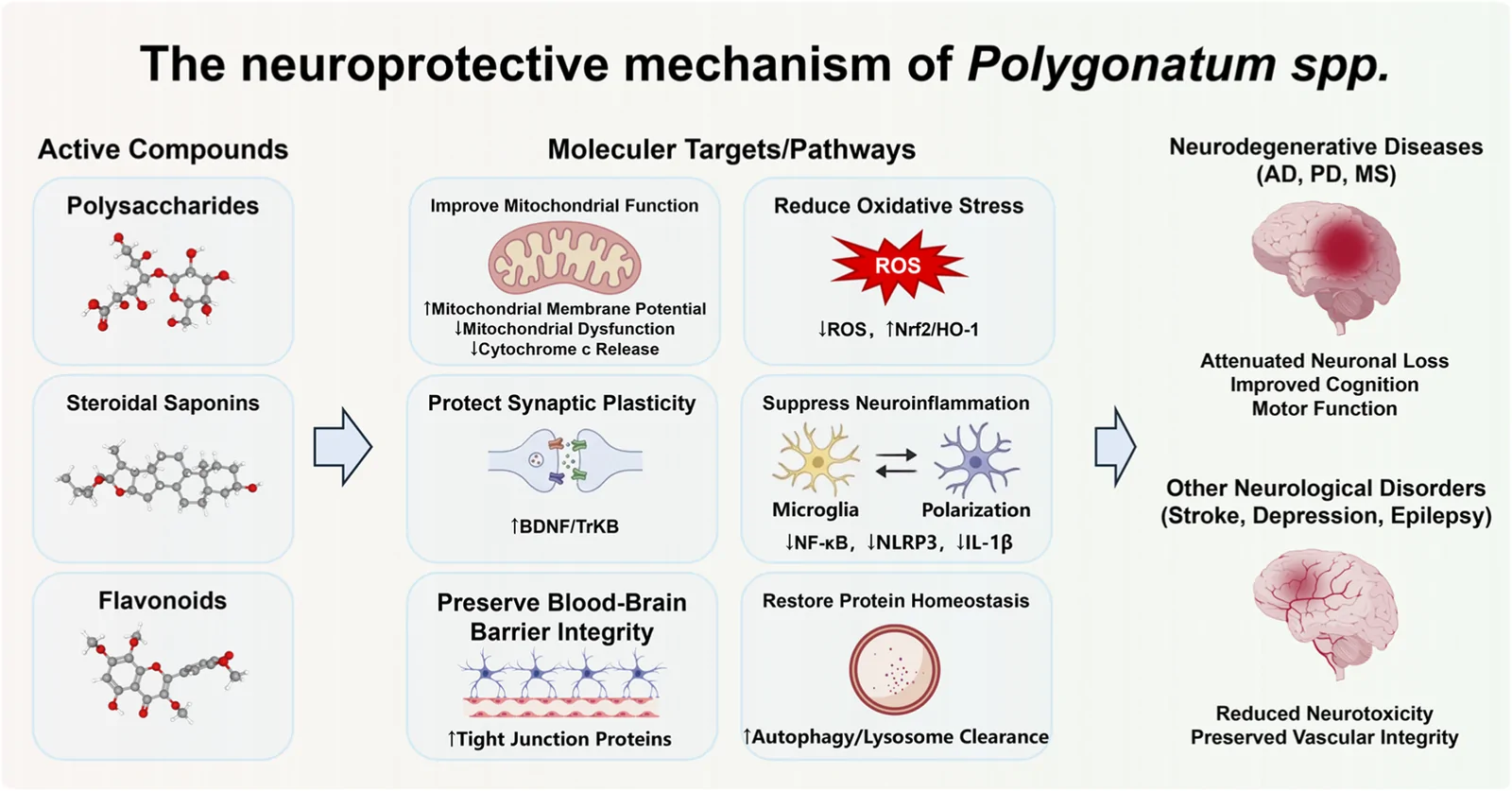
Proposed neuroprotective mechanisms of Polygonatum spp. Bioactive constituents, including polysaccharides, steroidal saponins, and flavonoids, exert neuroprotective effects by improving mitochondrial function, reducing oxidative stress, preserving synaptic plasticity, suppressing neuroinflammation, maintaining blood–brain barrier integrity, and restoring protein homeostasis, thereby protecting against neurodegenerative diseases (AD, PD, and MS) and other neurological.

Accordingly, this review systematically summarizes the botanical characteristics, phytochemical composition, and pharmacological activities of *Polygonatum*, with particular emphasis on the molecular mechanisms by which it modulates the functions of different glial cell populations. Specifically, we discuss the regulatory effects of *Polygonatum* on microglia, oligodendrocytes, neurons, and other neural cell types, and comprehensively summarize its neuroprotective actions and underlying molecular mechanisms in AD, PD, MS, stroke, and psychiatric disorders. This review aims to provide a comprehensive theoretical foundation and future research perspectives for the pharmacological development of *Polygonatum* and its clinical application in the prevention and treatment of neurodegenerative and other neurological diseases.

## 2 Literature search strategy

A literature search was conducted using PubMed, Web of Science, and Scopus to identify studies investigating the neuroprotective effects of *Polygonatum* spp. and their bioactive constituents. The search included combinations of the following terms: “Polygonatum”, “Polygonatum polysaccharides”, “Polygonatum sibiricum”, “Polygonatum cyrtonema”, “Polygonatum kingianum”, “neuroprotection”, “neurological disorders”, “microglia”, “astrocytes”, “oligodendrocytes”, “neurons”, “neuroinflammation”, “oxidative stress”, and “neurodegeneration”. Relevant studies published up to June 2026 were considered.

Original research articles, clinical studies, and high-quality reviews addressing the phytochemistry, pharmacological activities, cellular targets, or neurological effects of *Polygonatum* were included. Studies lacking sufficient methodological information, unrelated to neurological or glial mechanisms, or without accessible full text were excluded. Reference lists of relevant articles were additionally screened to identify potentially eligible studies. Because the present article was designed as a mechanistic narrative review rather than a formal systematic review, study selection was primarily guided by relevance to the cellular and molecular mechanisms discussed.

## 3 Botanical origin and phytochemical basis of *Polygonatum*

*Polygonatum* spp. are perennial herbaceous plants belonging to the genus *Polygonatum* of the family Asparagaceae. Their dried rhizomes have been extensively used as medicinal materials in China, Japan, Korea, and other regions of East and Southeast Asia for centuries (Chen et al., 2025; Li et al., 2024). In the Compendium of Materia Medica (Bencao Gangmu), *Polygonatum* is described as a superior medicinal herb with the traditional functions of replenishing qi, nourishing yin, replenishing essence and marrow, and promoting longevity (Li et al., 2024). The current edition of the Chinese Pharmacopoeia recognizes three official medicinal species: *Polygonatum* kingianum Coll. et Hemsl., *Polygonatum* sibiricum Red., and *Polygonatum* cyrtonema Hua. As a medicinal and edible homologous plant, *Polygonatum* has been widely employed in both TCM and folk medicine for the prevention and treatment of a variety of diseases (Han et al., 2025; Liu et al., 2025; Wen et al., 2024).

From the perspective of TCM, Polygonatum is characterized by the functions of replenishing qi, nourishing yin, strengthening the spleen, moistening the lung, and tonifying the kidney. Neurological and neuropsychiatric manifestations such as fatigue, cognitive decline, forgetfulness, sleep disturbance, and age-related functional deterioration are often interpreted within TCM as being associated, at least in part, with deficiency of qi and yin, insufficiency of essence and marrow, and dysfunction of the spleen and kidney. On this theoretical basis, Polygonatum has traditionally been used to replenish qi and nourish yin, enrich essence, and support systemic functional recovery. Although these traditional concepts do not directly correspond to modern neurobiological mechanisms, they provide the historical and theoretical rationale for the use of Polygonatum in conditions involving cognitive, behavioral, and age-related neurological dysfunction.

In clinical practice, Polygonatum is more commonly used as a component of multi-herb TCM formulations than as a single-agent therapy. Within the TCM framework, it is generally combined with other herbs according to specific syndrome patterns, particularly in conditions associated with fatigue, cognitive decline, sleep disturbance, and age-related functional deterioration. Such formulations are intended to achieve complementary effects through simultaneous regulation of multiple physiological systems rather than through a single molecular target. This multi-component therapeutic strategy is conceptually consistent with the emerging pharmacological evidence showing that Polygonatum can modulate oxidative stress, neuroinflammation, mitochondrial function, immune homeostasis, and the gut–brain axis. However, direct clinical evidence specifically evaluating Polygonatum-containing formulas in neurological disorders remains limited, and the independent contribution of Polygonatum within complex herbal combinations is often difficult to distinguish from that of other components. Therefore, future clinical studies using standardized Polygonatum-containing formulations are needed to clarify their therapeutic efficacy, optimal composition, and safety in neurological applications.

As a medicinal and edible homologous plant, Polygonatum also has potential advantages for daily health maintenance and long-term nutritional intervention. Compared with conventional pharmacological agents that are primarily used after disease onset, Polygonatum-based foods and dietary preparations may be more suitable for continuous intake in populations requiring nutritional support, fatigue management, healthy aging, or maintenance of metabolic and immune homeostasis. Its polysaccharides, flavonoids, and steroidal saponins provide a pharmacological basis for the development of functional foods and dietary supplements with antioxidant, anti-inflammatory, immunomodulatory, and metabolic regulatory properties. This medicinal–food dual-use characteristic may facilitate long-term health management and preventive applications. However, standardized preparation, dose control, quality evaluation, and long-term safety assessment remain necessary before such products can be recommended for specific neurological indications.

*Polygonatum* possesses a rich and diverse phytochemical profile, including polysaccharides, flavonoids, terpenoids, alkaloids, steroidal saponins, and trace elements, which has attracted increasing scientific attention in recent years (Li et al., 2024). Modern pharmacological studies have demonstrated that *Polygonatum* exhibits a broad spectrum of biological activities, including antioxidant, anti-inflammatory, immunomodulatory, hypoglycemic, and metabolic regulatory effects, while exhibiting minimal toxicity or adverse effects (Jiang et al., 2024; Ren et al., 2024; Zhang et al., 2022a). These pharmacological properties provide a strong scientific basis for its potential application in the prevention and treatment of neurodegenerative diseases. Chromatographic and mass spectrometric analyses have identified several major classes of bioactive constituents in *Polygonatum* rhizomes (Cao et al., 2025), and accumulating evidence indicates that these phytochemicals exhibit distinct yet complementary pharmacological activities in different neurodegenerative disease models (Table 1).

**TABLE 1:** Major bioactive constituents of *Polygonatum* and their neuroprotective mechanisms.

Bioactive constituent	Disease models	Characteristics and pharmacological activities	Major mechanisms
PSPs	AD(Chen et al., 2025), PD(Zhang et al., 2022), MS(Ren et al., 2024)	PSPs comprise a heterogeneous group of macromolecules with a broad molecular weight distribution, primarily including arabinogalactans, mannans, and pectin-like polysaccharides. They exhibit potent immunomodulatory and antioxidant activities and predominantly confer neuroprotection through the regulation of immune responses (Chen et al., 2025; Huang et al., 2025)	Activation of the Nrf2/HO-1 signaling pathway, regulation of Th17/Treg immune balance, reduction of reactive oxygen species (ROS), and enhancement of autophagy
Steroidal saponins	PD,MS(Ren et al., 2024), amyotrophic lateral sclerosis (ALS)	*Polygonatum* contains multiple steroidal saponins, including *Polygonatum* saponins A, B, and C, which possess anti-inflammatory and anti-apoptotic properties and primarily exert neuroprotective effects by suppressing neuronal apoptosis (Ren et al., 2024; Zheng et al., 2026)	Regulation of the NF-κB/MAPK and Bcl-2/Bax signaling pathways, thereby promoting neuronal survival (Zheng et al., 2026)
Flavonoids	AD, PD	*Polygonatum* flavonoids comprise flavones, isoflavones, flavanones, homoisoflavonoids, pterocarpans, and chalcones, which primarily exhibit antioxidant and anti-inflammatory activities (Cao et al., 2025; Tang et al., 2025; Zhang et al., 2023)	Scavenging ROS, reducing lipid peroxidation, and synergistically enhancing the neuroprotective effects of PSPs

It should be noted that Polygonatum polysaccharides (PSPs) do not represent a chemically uniform entity. Their molecular weight distribution and monosaccharide composition vary among P. kingianum, P. sibiricum, and P. cyrtonema, and may also be influenced by geographical origin, processing procedures, and extraction methods (Zhao et al., 2020). Such chemical heterogeneity may contribute to differences in biological activity and complicate direct comparisons across studies. Therefore, the pharmacological effects attributed to “PSPs” should be interpreted in the context of their specific botanical source and chemical characteristics. Representative chemical profiles of PSPs reported in the cited studies are summarized in Table 2.

**TABLE 2:** Representative chemical profiles of Polygonatum polysaccharides used in or relevant to the cited studies.

Species/PSP	Source and preparation	Molecular weight	Monosaccharide composition	Representative study/relevance
P. kingianum (PKP1)	Jianshui, Yunnan, China. Aqueous extraction followed by deproteinization, dialysis, ethanol precipitation, Cellulose DE-52 and Sephadex G-50 purification	Mw: 5.3 × 10^3^ Da PDI: 1.20	Predominantly fructose and glucose; Fru/Glc ≈27.86:1	Han et al., 2025. Anti-inflammatory activity and gut microbiota modulation
P. cyrtonema (PCP)	P. cyrtonema Hua. Sevage deproteinization followed by D101 macroporous resin purification; geographic origin not specified	Peak Mw: 2.489 kDa Mw: 2.51 kDa Mn: 1.473 kDa	Ara 1.04%; Rha 0.31%; Gal 10.38%; Glc 80.08%; Xyl 0.26%; Man 3.12%; GalA 3.98%; GulA 0.31%; GlcA 0.31%	Li et al., 2022. BV2 microglial oxidative stress and ferroptosis; NRF2/HO-1 pathway
P. sibiricum/Polygonati Rhizoma PSP	Crude polysaccharide preparation; detailed harvesting region not reported. Structural characterization by GPC-MALLS and ion chromatography	Mw: 20.48 kDa Mn: 2.18 kDa Mw/Mn: 9.394	Glucose, mannose and galactose; molar ratio 98.47:1.07:0.45	Zhang et al., 2024. CUMS-related depression model; microglial polarization and neuroinflammation
P. sibiricum/P. cyrtonema/P. kingianum	Comparative analysis of 14 batches from multiple sources using HPGPC, monosaccharide analysis and FTIR.	>4.1 × 10^5^ Da and 2.8–5.4 × 10^3^ Da	High-Mw fractions mainly pectic polysaccharides; low-Mw fractions mainly fructans. Profiles varied among species and batches	Comparative saccharide-mapping study. Demonstrates species- and batch-dependent PSP heterogeneity

PSPs, are chemically heterogeneous. Molecular-weight distribution and monosaccharide composition vary with botanical species, geographic origin, processing, extraction and purification. NR, not reported.

The processing methods used for *Polygonatum* markedly influence its phytochemical composition and biological activities (Cao et al., 2025). Moreover, differences in plant species, cultivation environments, and processing procedures can substantially alter both the chemical profile and pharmacological efficacy of *Polygonatum* preparations (Wang et al., 2025). Specifically, the traditional “nine steaming and nine sun-drying” process significantly enhances the solubility of polysaccharides and improves their antioxidant capacity (Cao et al., 2025; Zhang et al., 2023). In contrast, low-temperature drying better preserves flavonoid content, thereby minimizing the loss of biological activity (Cao et al., 2025; Zhang et al., 2023). Furthermore, extraction methods, such as aqueous or ethanol extraction, directly determine the yield of polysaccharides and steroidal saponins, resulting in considerable differences in the pharmacological potency of different extracts (Huang et al., 2025; Zheng et al., 2026). Notably, the bioactive constituents of *Polygonatum* exhibit complex synergistic interactions. Although individual compounds generally display limited pharmacological efficacy, combinations of multiple constituents can simultaneously modulate diverse molecular targets and signaling pathways through complementary mechanisms (Li et al., 2024; Xiao et al., 2022). This multi-component, multi-target, and multi-pathway mode of action provides a mechanistic basis for the superior therapeutic potential of natural products over conventional single-target drugs in the management of complex diseases (Figure 2).

**FIGURE 2:**
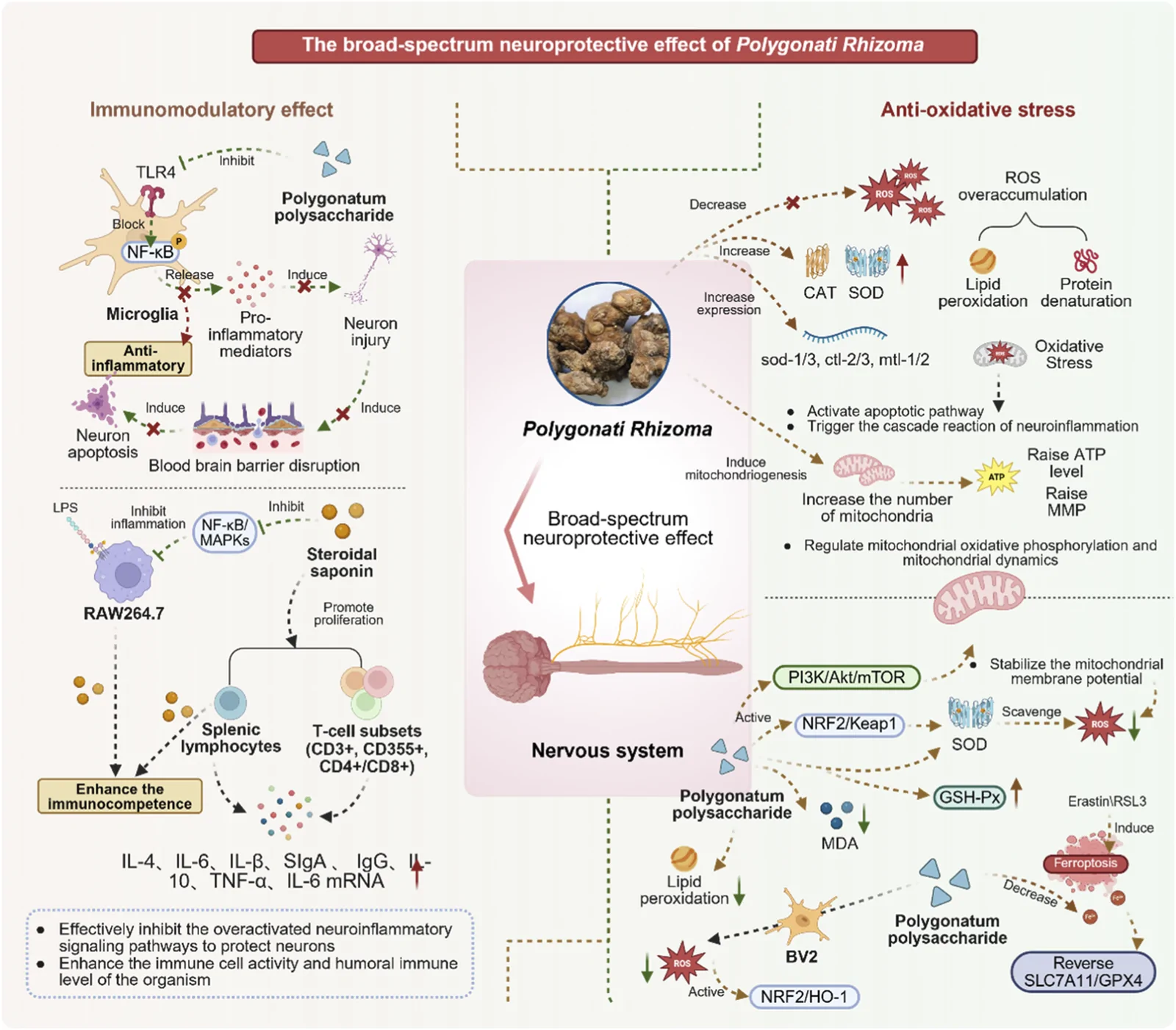
Schematic illustration of the broad-spectrum neuroprotective mechanisms of *Polygonati Rhizoma*. Its major bioactive constituents, including polysaccharides and steroidal saponins, regulate multiple pathogenic pathways involved in neurological disorders, including neuroinflammation, oxidative stress, mitochondrial dysfunction, ferroptosis, and immune responses, thereby promoting neuronal survival and maintaining nervous system homeostasis.

## 4 Broad-spectrum neuroprotective effects of *Polygonatum*

### 4.1 Antioxidant effects

#### 4.1.1 CNSis highly susceptible to oxidative stress

Owing to its high oxygen consumption, abundant lipid content, and relatively limited antioxidant defense capacity, excessive accumulation of ROS readily induces lipid peroxidation, protein oxidation, and cellular dysfunction (Cobley et al., 2018; Davì et al., 2025; Fernández-Bertólez et al., 2025; Lorca et al., 2025; Trubalski et al., 2025). Under pathological conditions, oxidative stress not only directly damages neuronal membranes and DNA but also activates mitochondrial apoptotic pathways and triggers neuroinflammatory cascades, thereby exacerbating neuronal injury (Ali et al., 2025; Choi et al., 2024; Gandhi and Abramov, 2012). Accumulating evidence indicates that oxidative stress represents a common early pathogenic event underlying numerous neurological disorders, including AD, PD, and stroke (Lin and Beal, 2006; Foret et al., 2024; Qin et al., 2025; Yutani et al., 2024). Trishna Debnath et al. demonstrated that aqueous extracts of PSPs possess potent antioxidant and anti-inflammatory activities, suggesting their potential application as natural antioxidant and anti-inflammatory agents in the food and pharmaceutical industries (Chen et al., 2025; Debnath et al., 2013; Wang et al., 2026; Zheng, 2020).

Extensive studies have shown that *Polygonatum* effectively attenuates oxidative stress in CNS disorders by reducing intracellular ROS levels, enhancing the activities of antioxidant enzymes such as superoxide dismutase (SOD) and catalase (CAT), and upregulating the mRNA expression of antioxidant genes, including SOD-1/3, CTL-2/3, and MTL-1/2. Moreover, *Polygonatum* promotes mitochondrial biogenesis, increases mitochondrial abundance, elevates intracellular ATP production and mitochondrial membrane potential (MMP), and regulates mitochondrial oxidative phosphorylation and mitochondrial dynamics, thereby alleviating the degeneration of GABAergic and serotonergic neurons (Lin et al., 2024; Wei et al., 2024; Zhang et al., 2022b).

PSPs further enhance endogenous antioxidant defenses by activating the NRF2/Keap1 signaling pathway, thereby increasing the expression and activity of antioxidant enzymes such as SOD, scavenging ROS, and suppressing lipid peroxidation. In addition, PSPs regulate the PI3K/Akt/mTOR signaling pathway to stabilize mitochondrial membrane potential, improve oxidative phosphorylation, and reduce mitochondrial ROS generation, thereby preserving mitochondrial function under oxidative stress conditions (Zhang et al., 2022a).

Experimental evidence further demonstrates that PSPs significantly reduce malondialdehyde (MDA) levels while increasing the activities of SOD and glutathione peroxidase (GSH-Px), accompanied by decreases in lipid peroxides and lipofuscin accumulation (Cui et al., 2018). In H2O2-induced BV2 microglial cells, PSPs attenuate intracellular ROS accumulation by activating the NRF2/HO-1 signaling pathway, resulting in the upregulation of antioxidant enzymes, including CAT, NAD(P)H quinone oxidoreductase 1 (NQO1), and glutathione peroxidase 1 (GPX1), while suppressing the expression of pro-oxidant enzymes such as acyl-CoA synthetase long-chain family member 4 (ACSL4), cyclooxygenase-2 (COX2), and NADPH oxidase 1 (NOX1). Consequently, oxidative stress-induced apoptosis is markedly alleviated. Furthermore, in erastin- or RSL3-induced ferroptosis models, PSPs reduce intracellular and mitochondrial Fe2+ accumulation, restore the expression of SLC7A11 and glutathione peroxidase 4 (GPX4), and re-establish glutathione homeostasis through activation of the NRF2/HO-1 pathway. Notably, these protective effects are selectively elicited under oxidative stress conditions without affecting normal cells, indicating a favorable safety profile (Li et al., 2022).

Collectively, *Polygonatum* exerts potent neuroprotective effects by simultaneously restoring antioxidant defense systems and maintaining mitochondrial homeostasis through multiple molecular targets. These coordinated actions effectively delay neuronal degeneration and provide a strong biological rationale for the therapeutic application of *Polygonatum* in CNS disorders.

### 4.2 Immunomodulatory effects

Increasing evidence has demonstrated that the polysaccharides, steroidal saponins, and flavonoids present in *Polygonatum* possess broad-spectrum immunomodulatory activities (Huang et al., 2025; Li et al., 2024). Among these, PSPs suppress inflammatory responses by regulating the Toll-like receptor 4 (TLR4)/myeloid differentiation primary response protein 88 (MyD88)/nuclear factor-κB (NF-κB) signaling pathway, thereby reducing the production of pro-inflammatory cytokines, including interleukin-6 (IL-6) and tumor necrosis factor-α (TNF-α) (Huang et al., 2025; Li et al., 2024). In addition, steroidal saponins isolated from *Polygonatum* inhibit lipopolysaccharide (LPS)-induced inflammatory responses in RAW264.7 macrophages by suppressing the NF-κB and mitogen-activated protein kinase (MAPK) signaling pathways, thereby exerting potent anti-inflammatory activity *in vitro* (Ren et al., 2024; Zheng et al., 2026).

Microglia are the resident immune cells of the CNS and serve as the first line of defense against pathogenic insults (Prinz et al., 2019). Upon activation by pathogens or damage-associated stimuli, TLR4 signaling triggers persistent activation of the NF-κB pathway, resulting in excessive production of pro-inflammatory mediators that ultimately contribute to neuronal injury (Glass et al., 2010; Kawai and Akira, 2010). This exaggerated inflammatory response not only facilitates pathogen clearance but also disrupts blood-brain barrier integrity and promotes neuronal apoptosis (Glass et al., 2010). PSPs significantly downregulate TLR4 expression in activated microglia, thereby inhibiting NF-κB p65 phosphorylation and suppressing the release of inflammatory cytokines such as TNF-α and IL-1β. Consequently, both the mRNA and protein expression levels of downstream pro-inflammatory mediators, including TNF-α, IL-1β, and IL-6, are markedly reduced, resulting in robust anti-inflammatory and neuroprotective effects (Zhang et al., 2022a).

Beyond suppressing neuroinflammation, *Polygonatum* also enhances systemic immune function. *Polygonatum* steroidal saponins stimulate the proliferation of splenic lymphocytes and T-cell subsets, including CD3^+^, CD4+/CD8+, and NK (CD355+) cells, while increasing serum lactate dehydrogenase and acid phosphatase activities. They also elevate the levels of cytokines and immunoglobulins, including interferon-γ (IFN-γ), IL-4, IL-6, IL-1β, secretory immunoglobulin A (SIgA), and immunoglobulin G (IgG), together with enhanced mRNA expression of IL-10, TNF-α, and IL-6 (Huang et al., 2025; Ren et al., 2024). Similarly, PSPs significantly enhance the immune activities of cultured murine splenic lymphocytes and macrophages *in vitro* (Chen et al., 2025; Zhang et al., 2022a).

Taken together, *Polygonatum* exhibits a bidirectional immunomodulatory profile. It suppresses excessive neuroinflammatory signaling to protect neurons while simultaneously enhancing systemic immune cell function and humoral immunity. These complementary activities underscore the unique therapeutic potential of *Polygonatum* as a multi-target immunomodulatory agent for the prevention and treatment of neurological diseases.

## 5 Neuroprotective effects of *Polygonatum* through the regulation of glial cell function

In recent years, glial cells have evolved from being regarded merely as structural support cells to becoming key therapeutic targets in neurological disease research (Gong et al., 2026). Among them, microglia are responsible for immune surveillance and inflammatory regulation within the central nervous system (CNS) (Cavalcanti et al., 2025); oligodendrocytes maintain myelin integrity and facilitate axonal impulse conduction (Simons and Nave, 2016); astrocytes contribute to blood-brain barrier homeostasis, neurotrophic support, and cerebral energy metabolism (Cheng et al., 2026; Dong et al., 2025); and oligodendrocyte precursor cells (OPCs), together with other newly identified glial populations, play indispensable roles in neural regeneration and tissue repair (Auguste et al., 2022). Increasing evidence demonstrates that *Polygonatum* and its bioactive constituents exert broad-spectrum neuroprotective effects by modulating the biological functions of distinct glial cell populations, thereby improving the neuroimmune microenvironment, promoting neuronal survival, and facilitating tissue repair in a variety of neurological disorders (Lu et al., 2026; Wu et al., 2025). Accordingly, this section summarizes the neuroprotective mechanisms of *Polygonatum* according to its primary glial cell targets (Figure 3).

**FIGURE 3:**
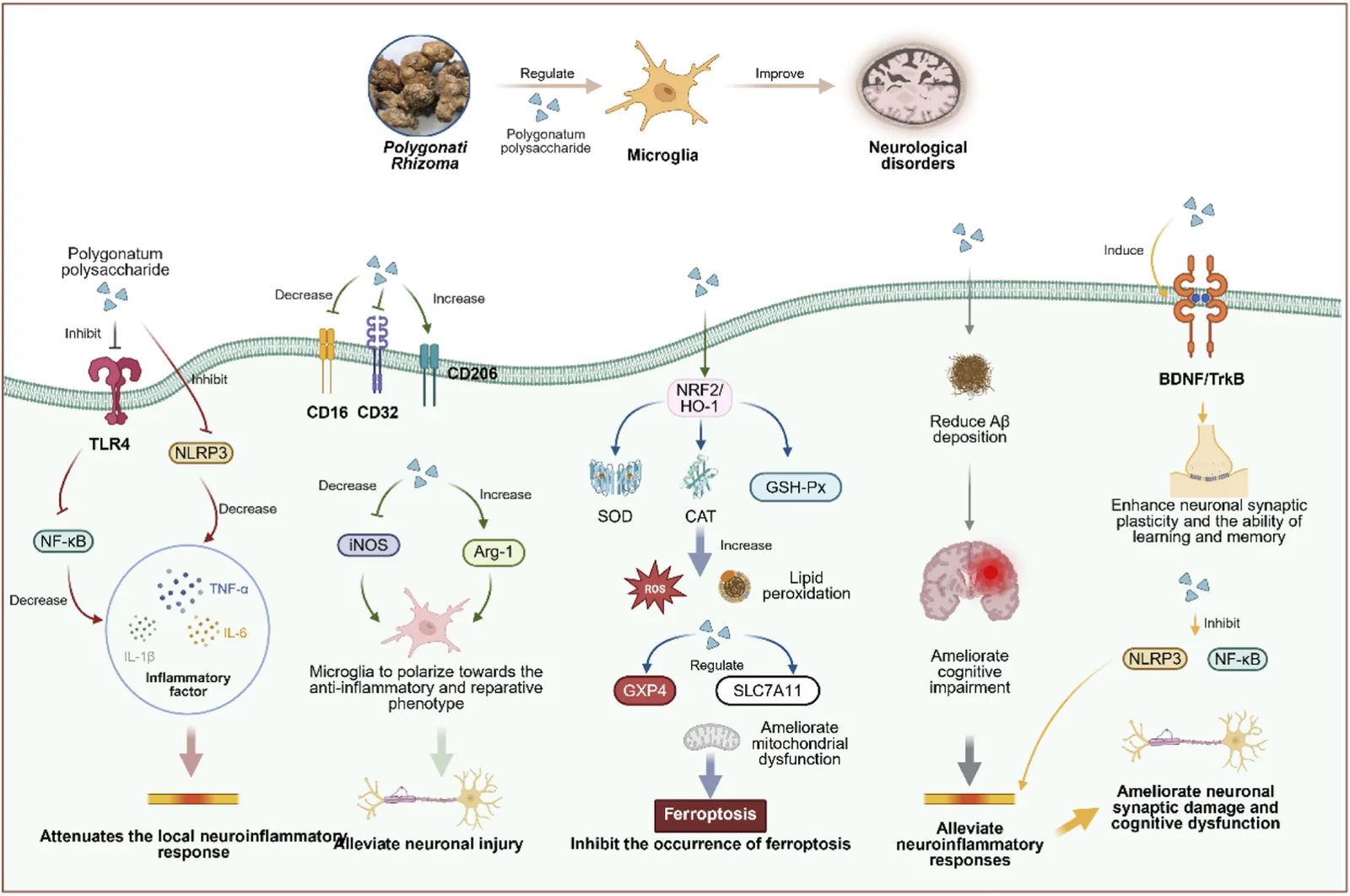
Proposed mechanisms underlying the neuroprotective effects of *Polygonatum* polysaccharides. *Polygonatum* polysaccharides alleviate neuroinflammation by inhibiting the TLR4/NF-κB/NLRP3 pathway and promoting anti-inflammatory microglial polarization, suppress ferroptosis through activation of the Nrf2/HO-1 antioxidant pathway, reduce Aβ deposition, enhance BDNF/TrkB-mediated synaptic plasticity, and improve mitochondrial function, thereby attenuating neuronal injury and cognitive impairment.

### 5.1 Regulation of microglial function in neurological diseases

#### 5.1.1 Suppression of aberrant microglial activation and neuroinflammation

Microglia are the resident immune cells of the CNS and play essential roles in maintaining neural homeostasis through immune surveillance, pathogen elimination, and phagocytic clearance of cellular debris (Chamkouri and Motlagh Mohavi, 2025; Lopez-Atalaya et al., 2018). However, in numerous neurological disorders, including AD, PD, depression, and traumatic brain injury, microglia undergo chronic aberrant activation and continuously release pro-inflammatory mediators, such as interleukin-1β (IL-1β), IL-6, tumor necrosis factor-α (TNF-α), and nitric oxide (NO). This persistent inflammatory response exacerbates neuronal injury and disrupts neural network function (Lull and Block, 2010; Zong et al., 2026). Consequently, suppressing excessive microglial activation has emerged as a promising strategy for neuroprotection (Noh et al., 2025; Patil et al., 2025).

PSPs have been shown to effectively modulate microglial inflammatory responses by suppressing the sustained activation of pro-inflammatory signaling pathways, thereby improving the neuroinflammatory microenvironment (Liu et al., 2023). In BV2 microglial cells, PSPs inhibit the activation of the Toll-like receptor 4 (TLR4)/nuclear factor-κB (NF-κB) signaling pathway and the NOD-like receptor family pyrin domain-containing 3 (NLRP3) inflammasome (Liu et al., 2022), resulting in significant reductions in the expression of IL-1β, TNF-α, and IL-6 and the subsequent release of inflammatory mediators. These effects contribute to the maintenance of microglial immune homeostasis (Li et al., 2022; Zhang et al., 2024). By attenuating local neuroinflammation, PSPs also reduce inflammation-induced secondary neuronal injury and establish a favorable cellular microenvironment for neural repair and functional recovery (Huang et al., 2021).

In AD models, PSPs suppress amyloid-β (Aβ)-induced microglial overactivation, attenuate chronic neuroinflammation, and improve cognitive dysfunction (Liu et al., 2025; Wang et al., 2026; Wu et al., 2025). Similarly, in depression models, PSPs alleviate chronic stress-induced microglial activation, reduce neuroinflammatory responses, and improve behavioral outcomes (Shen et al., 2021; Zhang et al., 2024). These findings suggest that microglia represent one of the principal cellular targets underlying the neuroprotective effects of *Polygonatum*.

#### 5.1.2 Regulation of microglial functional polarization to promote neural repair

Microglia exhibit remarkable functional plasticity and dynamically adapt their phenotypes in response to changes in the neural microenvironment (Sankowski and Prinz, 2025; Zong et al., 2026). Traditionally, activated microglia have been categorized into pro-inflammatory and reparative phenotypes (Chan et al., 2025). Pro-inflammatory microglia produce excessive inflammatory mediators and reactive oxygen species (ROS), thereby aggravating neuronal injury (Fan et al., 2023; Yang et al., 2025). In contrast, reparative microglia facilitate tissue repair, promote the clearance of abnormal protein aggregates, and contribute to the restoration of neural homeostasis (Gao et al., 2023; Yu et al., 2022). Therefore, promoting the transition of microglia from a pro-inflammatory to a reparative state is considered an important therapeutic strategy for neurological diseases (Wickstead et al., 2023).

PSPs effectively regulate microglial functional polarization (Liu et al., 2023). In both chronic unpredictable mild stress (CUMS)-induced depression models and BV2 microglial cells, PSPs significantly decrease the expression of pro-inflammatory markers, including CD16, CD32, and inducible nitric oxide synthase (iNOS), while simultaneously increasing the expression of reparative markers such as arginase-1 (Arg-1) and CD206, thereby promoting a shift toward an anti-inflammatory and tissue-repairing phenotype (Zhang et al., 2024). This phenotypic transition is accompanied by reduced neuroinflammation, increased expression of neurotrophic factors, attenuation of neuronal injury, and subsequent improvements in learning, memory, and depression-like behaviors (Xue et al., 2025).

Notably, recent studies have demonstrated that microglial functional states are considerably more heterogeneous than the traditional M1/M2 classification suggests. Nevertheless, substantial experimental evidence supports the ability of *Polygonatum* to promote the transition of microglia from inflammatory toward reparative functional states (Lu et al., 2026; Qin et al., 2023). Accordingly, interpreting the neuroprotective actions of *Polygonatum* from the perspective of microglial functional reprogramming rather than the simplistic M1/M2 paradigm is more consistent with current advances in neuroimmunology (Lan et al., 2026; Yu et al., 2025).

#### 5.1.3 Attenuation of oxidative stress and ferroptosis in microglia

In addition to inflammation, oxidative stress is a major contributor to microglial dysfunction (Geng et al., 2020; Simpson and Oliver, 2020). Persistent accumulation of ROS not only directly damages mitochondria but also induces lipid peroxidation and ferroptosis, thereby amplifying neuroinflammation and establishing a vicious cycle between oxidative stress and inflammatory signaling (Cheng et al., 2021; Ren et al., 2026).

Studies have demonstrated that PSPs markedly alleviate oxidative stress in BV2 microglia by activating the NRF2/HO-1 signaling pathway, reducing ROS production and lipid peroxidation, and enhancing the activities of antioxidant enzymes, including superoxide dismutase (SOD), catalase (CAT), and glutathione peroxidase (GSH-Px), thereby preserving normal microglial function (Debnath et al., 2013; Teng et al., 2023). Furthermore, PSPs regulate the expression of ferroptosis-associated proteins, including glutathione peroxidase 4 (GPX4) and solute carrier family seven member 11 (SLC7A11), improve mitochondrial dysfunction, attenuate lipid peroxidation, and effectively inhibit ferroptotic cell death in microglia (Li et al., 2022).

As oxidative injury is alleviated, the production of pro-inflammatory cytokines is substantially reduced, while the neuronal microenvironment is correspondingly improved, ultimately contributing to neuroprotection (Gao et al., 2023; Gui et al., 2024). These findings indicate that the maintenance of microglial redox homeostasis represents a key molecular mechanism underlying the neuroprotective effects of *Polygonatum* (Wu et al., 2025).

#### 5.1.4 Regulation of microglial phagocytosis and clearance of pathological protein aggregates

In addition to regulating neuroinflammation, microglia are the principal immune surveillance and phagocytic cells of the CNS (Li et al., 2026; Prinz et al., 2021). Under physiological conditions, they efficiently eliminate apoptotic cells, cellular debris, and aberrantly aggregated proteins, thereby maintaining brain homeostasis. However, during the progression of neurodegenerative diseases, microglial phagocytic capacity gradually declines, leading to the accumulation of amyloid-β (Aβ), hyperphosphorylated tau, and other metabolic waste products. This impaired clearance further aggravates neuroinflammation and neuronal degeneration. Consequently, restoring normal microglial phagocytic function has emerged as a promising therapeutic strategy for delaying neurodegenerative disease progression (Hampel et al., 2021; Liu et al., 2025; Scheltens et al., 2021; Xu et al., 2025; Zhang et al., 2026; Zhang et al., 2023).

Although direct evidence demonstrating that *Polygonatum* enhances microglial phagocytosis remains limited, available studies suggest that PSPs preserve normal microglial biological function by alleviating inflammation and oxidative stress, thereby facilitating the clearance of abnormal protein aggregates and cellular debris (Jiang et al., 2024). Consistent with this hypothesis, PSP treatment significantly reduces Aβ deposition, ameliorates cognitive impairment, and suppresses neuroinflammation in experimental models of AD (Liu et al., 2025; Luo et al., 2022; Wang et al., 2026; Zhang et al., 2026). Future investigations employing advanced approaches, particularly single-cell transcriptomics, are warranted to elucidate the molecular mechanisms by which *Polygonatum* regulates microglial phagocytic activity.

#### 5.1.5 Modulation of microglia-neuron interactions

Emerging evidence indicates that microglia function not only as immune effector cells but also as critical regulators of neuronal network homeostasis (Di Pietro et al., 2026). Under physiological conditions, microglia support neuronal survival by secreting neurotrophic factors, including brain-derived neurotrophic factor (BDNF) and transforming growth factor-β (TGF-β), while participating in synaptic pruning and neural circuit remodeling (Huang et al., 2021; Prowse and Hayley, 2021). Conversely, under pathological conditions, chronically activated microglia release excessive inflammatory cytokines and ROS, leading to mitochondrial dysfunction, synaptic damage, and neuronal apoptosis, thereby exacerbating neurological deficits (Gao et al., 2023).

Accumulating evidence suggests that PSPs optimize the neuronal microenvironment by suppressing microglial inflammatory activation and oxidative stress. These effects promote the activation of neurotrophic signaling pathways, including the BDNF/tropomyosin receptor kinase B (TrkB) pathway, thereby enhancing synaptic plasticity and improving learning and memory performance (Jiang et al., 2024; Wang et al., 2026; Zhang et al., 2022a). Moreover, in models of chronic stress- and simulated microgravity-induced cognitive impairment, PSPs attenuate microglia-mediated neuroinflammation by inhibiting activation of the NLRP3/NF-κB signaling pathway, resulting in reduced synaptic injury and improved cognitive function (Chen et al., 2025). Collectively, these findings indicate that *Polygonatum* not only directly regulates microglial function but also preserves neural network homeostasis by modulating reciprocal interactions between microglia and neurons, thereby contributing to its broad-spectrum neuroprotective effects.

### 5.2 Regulation of oligodendrocyte function

#### 5.2.1 *Polygonatum* attenuates the pathological changes associated with demyelinating diseases

Oligodendrocytes are the myelinating cells of the central nervous system (CNS) and are indispensable for the formation and maintenance of myelin sheaths. Dysfunction or loss of oligodendrocytes disrupts saltatory nerve conduction and represents a fundamental pathological feature of demyelinating disorders, particularly MS (Pajevic et al., 2023; Simkins et al., 2021; Xiang et al., 2026). In recent years, therapeutic strategies for MS have gradually shifted from solely suppressing immune-mediated inflammation toward promoting remyelination and functional neurological recovery. Consequently, preserving oligodendrocyte viability and function has become a major focus of neuroprotective research (Thalib et al., 2026; Zhang et al., 2011).

Although studies investigating the therapeutic effects of *Polygonatum* in MS remain limited, available experimental evidence suggests that PSPs effectively attenuate neuroinflammation, improve neurological function scores, and reduce the expression of inflammation-associated cytokines in experimental models, thereby delaying disease progression (Zhang et al., 2025). These findings indicate that *Polygonatum* possesses considerable therapeutic potential for the treatment of demyelinating disorders and provide an experimental basis for its future application in MS and related diseases.

#### 5.2.2 *Polygonatum* improves the inflammatory microenvironment to facilitate neurological recovery

The initiation and progression of MS are characterized by persistent neuroinflammation, during which excessive inflammatory mediators exacerbate neural tissue injury and impede functional recovery (Simkins et al., 2021). Therefore, alleviating neuroinflammation remains one of the principal therapeutic strategies for MS management (Boutitah-Benyaich et al., 2025).

Current studies have demonstrated that PSPs significantly reduce the expression of pro-inflammatory cytokines, attenuate CNS inflammatory responses, and improve neurological deficits in experimental animals (Zhang et al., 2025). However, direct evidence regarding the regulation of oligodendrocyte biology by *Polygonatum* remains limited. Consequently, most existing studies have focused on its overall anti-inflammatory and neuroprotective properties rather than its direct effects on oligodendrocytes (Lu et al., 2026). Future investigations are warranted to determine whether *Polygonatum* directly regulates oligodendrocyte survival, maturation, remyelination, and myelin repair (Figure 4).

**FIGURE 4:**
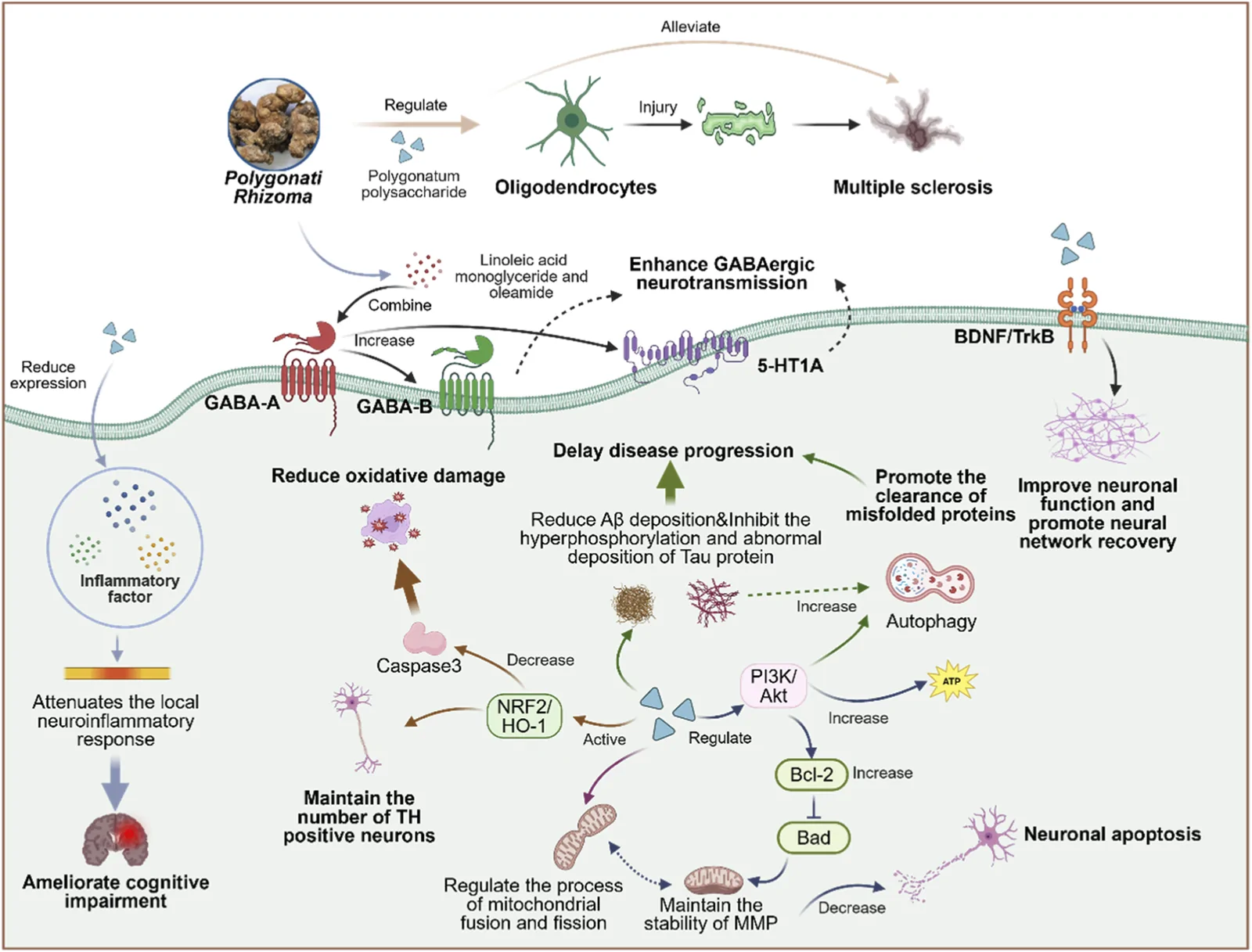
Schematic illustration of the neuroprotective mechanisms of *Polygonatum* polysaccharides. *Polygonatum* polysaccharides exert neuroprotective effects by modulating neurotransmitter signaling, suppressing neuroinflammation and oxidative stress, preserving mitochondrial function, promoting autophagy, reducing Aβ and tau pathology, and enhancing BDNF/TrkB-mediated neuronal plasticity, thereby delaying neurodegeneration and improving cognitive function.

### 5.3 Regulation of neuronal function

Neurons are the fundamental structural and functional units responsible for information processing and transmission within the CNS. Neuronal dysfunction and degeneration are central pathological features shared by numerous neurological disorders, including AD, PD, stroke, post-traumatic stress disorder (PTSD), and age-related cognitive impairment (Li et al., 2014). Increasing evidence indicates that *Polygonatum* and its bioactive constituents exert neuroprotective effects through multiple mechanisms, including improving neuronal energy metabolism, suppressing pathological protein aggregation, preserving mitochondrial homeostasis, enhancing synaptic plasticity, and restoring neurotransmitter balance (Lu et al., 2026; Wu et al., 2025).

#### 5.3.1 Inhibition of pathological protein aggregation

Aberrant protein aggregation is a hallmark of AD pathology. The accumulation of amyloid-β (Aβ) peptides and hyperphosphorylated tau protein progressively impairs neuronal structure and function, ultimately leading to cognitive decline (Hampel et al., 2021; Scheltens et al., 2021; Zhang et al., 2023). PSPs have been shown to effectively alleviate Aβ-induced neurotoxicity. In a *Caenorhabditis elegans* model of AD, PSP treatment significantly prolonged lifespan, enhanced antioxidant capacity, reduced Aβ aggregation, improved neuromuscular dysfunction, and inhibited tau hyperphosphorylation and abnormal protein deposition, thereby delaying disease progression (Liu et al., 2025). Furthermore, in AD mouse models, PSPs regulate the phosphatidylinositol 3-kinase/protein kinase B (PI3K/Akt) signaling pathway, reduce tau deposition in the brain, suppress the expression of pro-inflammatory cytokines, and alleviate neuronal pathological damage (Zhang et al., 2026).

Collectively, current evidence demonstrates that *Polygonatum* inhibits both Aβ aggregation and tau hyperphosphorylation, providing experimental support for its protective effects against neuronal degeneration.

#### 5.3.2 Improvement of mitochondrial function and energy metabolism

Mitochondrial dysfunction is a common pathological feature of numerous neurological diseases and contributes to impaired ATP production, excessive ROS generation, and neuronal death (Norat et al., 2020). PSPs activate the PI3K/Akt signaling pathway to enhance neuronal glucose utilization, increase ATP production and mitochondrial membrane potential, and regulate mitochondrial fusion and fission dynamics, thereby maintaining mitochondrial homeostasis and improving neuronal energy metabolism (Luo et al., 2022; Zhang et al., 2022b). In addition, in experimental models of PD, PSPs activate the sirtuin 1 (Sirt1)/nuclear factor erythroid 2-related factor 2 (Nrf2) signaling axis, suppress caspase-3 activation, attenuate oxidative damage, and protect dopaminergic neurons in the substantia nigra (Zhang et al., 2022a).

#### 5.3.3 Regulation of autophagy to maintain proteostasis

The autophagy-lysosomal system is essential for maintaining neuronal proteostasis, and its dysfunction leads to the accumulation of misfolded proteins, thereby accelerating neurodegeneration (Wang et al., 2010). PSPs regulate the PI3K/Akt signaling pathway in a leptin receptor (LepR)-dependent manner, thereby restoring autophagic activity, promoting the clearance of misfolded proteins, reducing abnormal tau accumulation, and preserving synaptic proteins and neuronal structural integrity (Zhang et al., 2026).

These findings suggest that modulation of neuronal autophagy represents an important mechanism underlying the neuroprotective effects of *Polygonatum* against neurodegenerative diseases.

#### 5.3.4 Inhibition of neuronal apoptosis

Progressive neuronal loss is a pathological hallmark common to many neurological disorders (Lin et al., 2025). In animal models of PD, PSPs activate the Sirt1/Nrf2 signaling axis to suppress caspase-3 expression, reduce apoptosis of dopaminergic neurons, and preserve the number of tyrosine hydroxylase (TH)-positive neurons (Zhang et al., 2022a). In cerebral ischemia-reperfusion models, bioactive constituents of *Polygonatum* activate the PI3K/Akt signaling pathway, increase B-cell lymphoma 2 (Bcl-2) expression, inhibit Bcl-2-associated agonist of cell death (Bad) activation, and maintain mitochondrial membrane potential, thereby reducing programmed neuronal cell death (Li et al., 2022). Furthermore, in experimental intracerebral hemorrhage models, *Polygonatum* regulates the Bcl-2/Bax ratio, attenuates neuronal apoptosis in perihematomal regions, and reduces secondary brain injury (Zhang et al., 2015).

These findings collectively indicate that inhibition of neuronal apoptosis constitutes one of the principal mechanisms responsible for the neuroprotective effects of *Polygonatum*.

#### 5.3.5 Enhancement of synaptic plasticity

Synaptic dysfunction is a major pathological basis of cognitive impairment. *Polygonatum* enhances the expression of neurotrophic factors by activating the brain-derived neurotrophic factor (BDNF)/tropomyosin receptor kinase B (TrkB) signaling pathway, thereby improving synaptic ultrastructure and promoting the recovery of learning and memory functions. Following treatment, experimental animals exhibit significantly improved motor coordination and spontaneous locomotor activity, accompanied by increased densities of TH-positive nerve fibers in the striatum, indicating that *Polygonatum* effectively improves neuronal function and facilitates neural network reconstruction (Zhang et al., 2022a).

#### 5.3.6 Restoration of neurotransmitter homeostasis

Neurotransmitter imbalance is a common pathological feature of numerous neuropsychiatric disorders, including depression, anxiety, insomnia, and epilepsy. PSPs increase cerebral levels of serotonin (5-HT) and dopamine (DA), alleviate stress-induced neurotransmitter dysregulation, and improve neuroendocrine function through modulation of the hypothalamic-pituitary-adrenal (HPA) axis (Shen et al., 2021; Yang et al., 2024; Zhang et al., 2023). Furthermore, 2-monoolein linoleate and oleamide, two bioactive compounds isolated from *Polygonatum* rhizomes, interact with γ-aminobutyric acid type A (GABAA) receptors, upregulate the expression of GABAA receptor subunit α2 (GABAA-R2) and 5-hydroxytryptamine receptor 1A (5-HT1A), and enhance GABAergic neurotransmission, thereby exerting sedative and antiepileptic effects (Goldschen-Ohm, 2022).

Clinical studies have further demonstrated that *Polygonatum* rhizome extract improves sleep quality, prolongs total sleep duration, and increases perfusion within the medial prefrontal cortex in patients with mild insomnia, providing preliminary clinical evidence supporting its beneficial effects on neurological function (Ha et al., 2019).

### 5.4 Regulation of astrocytes and glial cell interactions

In addition to microglia and oligodendrocytes, increasing evidence indicates that oligodendrocyte precursor cells (OPCs), astrocytes, and other glial cell subpopulations also play critical roles in the pathogenesis and progression of neurological diseases (Li et al., 2025). These cell types collectively maintain central nervous system (CNS) homeostasis by secreting neurotrophic factors, regulating ionic homeostasis, and preserving blood-brain barrier integrity. However, studies directly investigating the regulatory effects of *Polygonatum* on these glial populations remain limited. Current evidence has primarily focused on its overall neuroprotective and anti-neuroinflammatory activities, and it remains unclear whether *Polygonatum* directly modulates the biological functions of astrocytes, OPCs, or other glial subtypes. Therefore, elucidating the cell type-specific mechanisms of *Polygonatum* in diverse glial populations represents an important direction for future investigation.

### 5.5 Regulation of other neural cell types and multicellular interactions

The pathogenesis of neurological diseases extends beyond neurons and glial cells and is frequently accompanied by dysfunction of cerebrovascular endothelial cells, disruption of blood-brain barrier integrity, and abnormalities in neuroendocrine regulation. Consequently, the neuroprotective effects of *Polygonatum* are likely mediated through coordinated regulation of multiple cell types and tissue systems.

In experimental models of cerebral ischemia-reperfusion injury and intracerebral hemorrhage, *Polygonatum* attenuates oxidative stress, suppresses neuroinflammation, and preserves blood-brain barrier integrity, thereby reducing cerebral edema and secondary brain injury (Li et al., 2025a; “Neuroprotective Effects of Polygona-Polysaccharose in Treatment of Ischemic Reperfusion Injury in Rats, 2018; Xu et al., 2025; Xue et al., 2025; Zhang et al., 2015). These findings suggest that the neuroprotective actions of *Polygonatum* are not restricted to neurons and glial cells but also involve improvement of the overall cerebral microenvironment.

In addition, *Polygonatum* exerts beneficial effects on neuroendocrine regulation in neuropsychiatric disorders. In models of chronic sleep deprivation (CSD) and chronic restraint stress (CRS), PSPs significantly reduce serum corticosterone (CORT) levels, restore the negative feedback regulation of the hypothalamic-pituitary-adrenal (HPA) axis, and attenuate inflammatory injury in the hippocampus (Shen et al., 2022; Yang et al., 2024). These findings indicate that *Polygonatum* may ameliorate stress-related neurological dysfunction by maintaining neuroendocrine homeostasis. Consistent with these experimental observations, a randomized, double-blind, placebo-controlled clinical trial demonstrated that 4 weeks of treatment with *Polygonatum* rhizome extract significantly improved Athens Insomnia Scale (AIS) scores, prolonged total sleep duration, and increased perfusion within the medial prefrontal cortex of the default mode network in patients with mild insomnia, providing clinical evidence supporting its therapeutic potential for sleep disorders (Lorca et al., 2025).

Furthermore, in experimental models of anxiety and post-traumatic stress disorder (PTSD), *Polygonatum* alleviates anxiety-like behaviors and abnormal fear memory while reducing oxidative stress and neuroinflammation (Xie et al., 2024; Zhang et al., 2026). In toxin-induced models of neurological injury, PSPs decrease the production of inflammatory cytokines, including interleukin-1β (IL-1β) and tumor necrosis factor-α (TNF-α), thereby attenuating neural tissue damage (Ho et al., 2015; Xie et al., 2024; Zhang et al., 2022a). Nevertheless, as most available studies have been conducted in whole-animal models, the specific target cell populations and molecular mechanisms underlying these protective effects remain to be elucidated (Figure 5).

**FIGURE 5:**
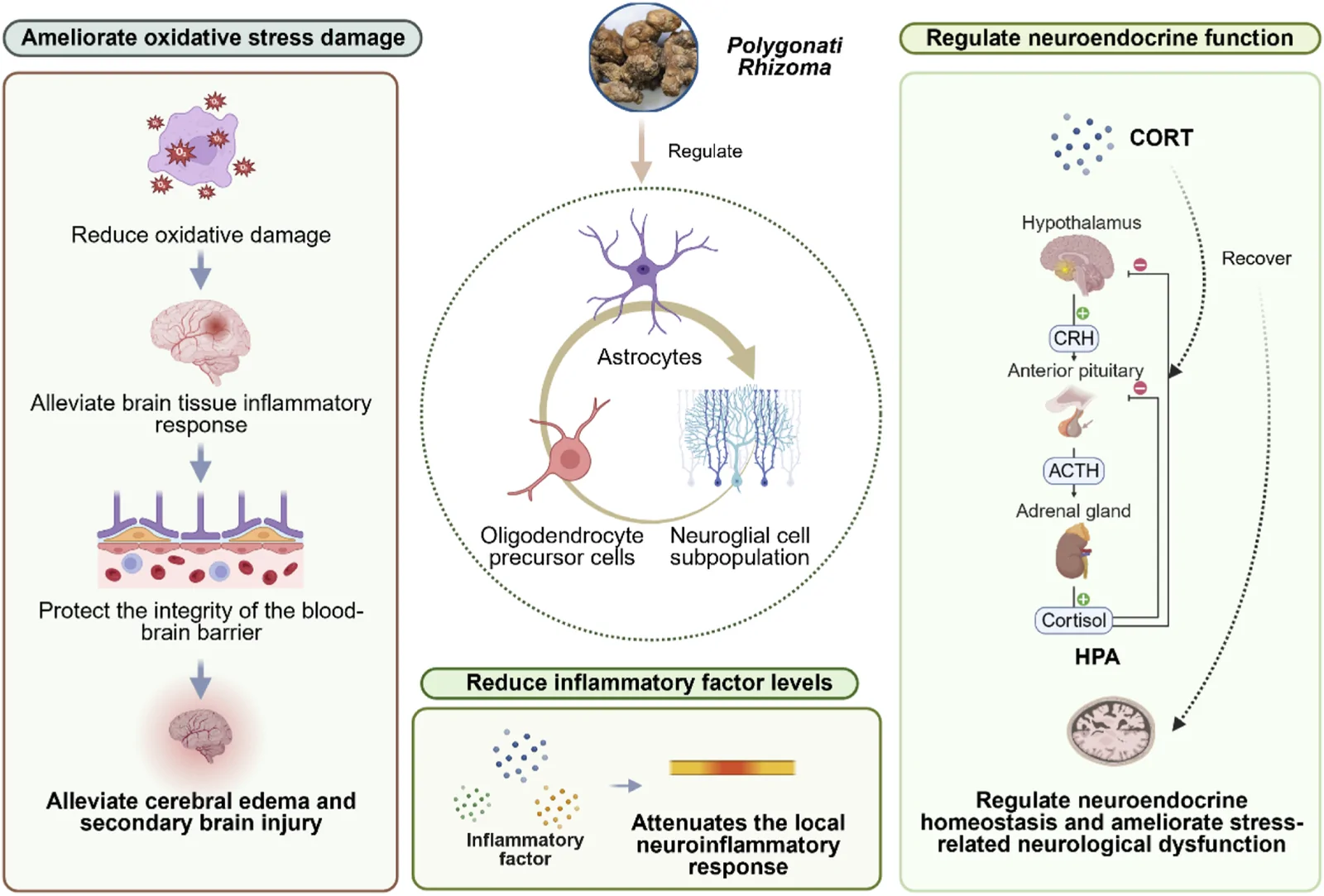
Schematic illustration of the neuroprotective effects of *Polygonati Rhizoma*. *Polygonati Rhizoma* exerts neuroprotective effects by reducing oxidative stress and neuroinflammation, preserving blood–brain barrier integrity, maintaining neural cell homeostasis, and regulating the hypothalamic–pituitary–adrenal (HPA) axis, thereby alleviating neurological dysfunction.

## 6 Safety, dosage, and translational considerations

The doses of Polygonatum-derived preparations used in experimental studies vary considerably depending on the botanical source, extraction procedure, active constituent, disease model, and route of administration. In neurological and related experimental models, Polygonatum polysaccharides (PSPs) are commonly administered orally within a range of approximately 45–400 mg/kg/day. For example, PSPs have been administered at 45 mg/kg/day in long-term mouse studies, whereas doses of 100, 200, and 400 mg/kg have been used in models of cognitive impairment. Notably, a dose of 400 mg/kg in mice corresponds to a human equivalent dose of approximately 32 mg/kg based on body surface area conversion, equivalent to approximately 2.1 g/day for a 65-kg adult. This suggests that some experimentally effective doses may be achievable through concentrated dietary supplements or standardized preparations, although direct extrapolation from animal dosing to human intake should be interpreted cautiously because differences in absorption, metabolism, bioavailability, and chemical composition may substantially influence systemic exposure (Huang et al., 2021; Chen et al., 2025).

From a safety perspective, available evidence generally suggests that PSPs have a relatively favorable tolerability profile at commonly used experimental doses. In mice, oral administration of PSPs at 45 mg/kg/day for 12 consecutive weeks did not significantly affect body weight, hematological parameters, or the histological appearance of the brain, liver, and kidney (Huang et al., 2021). Nevertheless, safety cannot be generalized across all Polygonatum preparations. Recent toxicological evidence indicates that excessive exposure to ethanolic extracts of P. sibiricum may induce behavioral abnormalities, chromosomal damage, biochemical disturbances, and structural injury in the liver, kidney, and spleen despite the absence of acute lethality. These findings emphasize that the toxicological profile may depend strongly on plant species, extraction method, chemical composition, dose, and treatment duration. Therefore, systematic studies defining the no-observed-adverse-effect level, maximum tolerated dose, chronic toxicity, genotoxicity, reproductive toxicity, and potential herb–drug interactions are still required before long-term clinical use can be adequately evaluated.

## 7 Conclusion and future perspectives

As a traditional tonic medicinal herb, *Polygonatum* has attracted increasing attention for its therapeutic potential across a broad spectrum of neurological disorders. Current evidence indicates that its bioactive constituents exert neuroprotective effects primarily by targeting multiple pathological processes, including oxidative stress, neuroinflammation, mitochondrial dysfunction, disruption of protein homeostasis, and blood-brain barrier impairment. To date, substantial evidence has demonstrated the multi-target neuroprotective potential of *Polygonatum* in AD, PD, and ischemic stroke. In contrast, research on MS, depression, epilepsy, and other neurological disorders remains largely at the mechanistic exploration stage, while direct evidence supporting its efficacy in autoimmune encephalitis, psychiatric disorders, and neuroinfectious diseases is still limited.

Future investigations should focus on elucidating the precise molecular and cellular mechanisms underlying the neuroprotective effects of *Polygonatum*, establishing standardized quality control systems for its bioactive constituents and preparations, and promoting high-quality clinical translation through well-designed preclinical and clinical studies. Furthermore, advances in single-cell transcriptomics, spatial omics, multi-omics integration, and artificial intelligence-assisted drug discovery are expected to facilitate the identification of cell type-specific targets and signaling networks regulated by *Polygonatum*. These efforts will provide a stronger scientific foundation for the development of *Polygonatum* as a standardized, evidence-based natural therapeutic resource for the prevention and treatment of neurological diseases and may ultimately contribute to addressing the growing global burden of neurological disorders.

Importantly, the present review differs from previous summaries of Polygonatum by adopting a cell-centered mechanistic framework. Rather than organizing the available evidence primarily according to individual neurological diseases or isolated pharmacological effects, we integrate the actions of Polygonatum across microglia, oligodendrocytes, astrocytes, neurons, and other components of the neural microenvironment. This perspective suggests that the neuroprotective effects of Polygonatum are unlikely to depend on a single molecular target, but may instead arise from coordinated regulation of neuroinflammation, redox homeostasis, mitochondrial function, proteostasis, synaptic integrity, and intercellular communication. In cell populations for which direct evidence remains limited, particularly astrocytes, oligodendrocytes, and oligodendrocyte precursor cells, signaling pathways such as TGF-β, Notch, PI3K/Akt, NRF2, and NF-κB may represent plausible mechanistic candidates based on the broader pharmacological actions of Polygonatum, although these possibilities require direct experimental validation. By integrating established evidence with these cell-specific mechanistic hypotheses, this review provides a broader conceptual framework for understanding Polygonatum as a potential regulator of multicellular homeostasis in neurological disorders.

Several important knowledge gaps nevertheless remain. Current mechanistic evidence is heavily concentrated on microglia and neurons, whereas direct studies of astrocytes, oligodendrocytes, oligodendrocyte precursor cells, and neurovascular cell populations remain scarce. In addition, many reported effects are derived from whole-animal or nonspecific cellular models, making it difficult to distinguish primary cellular targets from secondary responses to changes in the surrounding microenvironment. Considerable heterogeneity in Polygonatum species, extraction procedures, chemical composition, and experimental dosing further limits direct comparison across studies. Future investigations should therefore prioritize standardized preparations, cell type-specific experimental models, and advanced approaches such as single-cell transcriptomics, spatial omics, and integrated multi-omics analyses. Such strategies will be essential for identifying the principal responsive cell populations, establishing causal molecular pathways, and determining which biological effects are most relevant for clinical translation.

Another important translational gap concerns whether the neuroprotective effects of Polygonatum arise from direct actions within the central nervous system or indirectly through the gut–brain axis. Orally administered Polygonatum polysaccharides may influence gut microbial composition and microbial metabolism, thereby modulating systemic immune and metabolic signaling that ultimately affects brain function. At the same time, the extent to which intact bioactive constituents or microbiota-derived metabolites enter the circulation and cross the blood–brain barrier remains unclear. Therefore, future studies should integrate pharmacokinetic analysis, microbiome profiling, metabolomics, and blood–brain barrier permeability assessments to determine the relative contributions of direct central effects and microbiota-mediated indirect mechanisms.

Beyond these mechanistic uncertainties, an important issue for clinical translation is whether the neuroprotective effects of Polygonatum result from direct actions within the central nervous system or are mediated indirectly through the gut–brain axis. Orally administered Polygonatum polysaccharides may alter gut microbial composition and microbial metabolism, thereby influencing systemic immune and metabolic signaling with downstream effects on brain function. However, it remains unclear to what extent intact bioactive constituents or microbiota-derived metabolites enter the circulation and penetrate the blood–brain barrier. This uncertainty limits current interpretation of whether the observed neurological benefits primarily reflect direct central exposure or indirect microbiota-mediated mechanisms. Future studies combining pharmacokinetic analysis, microbiome profiling, metabolomics, and blood–brain barrier permeability assessment will therefore be essential to clarify the fate of orally administered Polygonatum constituents and to define the relative contribution of direct and gut–brain axis-mediated neuroprotective effects.
